# Hyperornithinemia‐hyperammonemia‐homocitrullinuria syndrome in pregnancy: Considerations for management and review of the literature

**DOI:** 10.1002/jmd2.12025

**Published:** 2019-03-14

**Authors:** Bernice Ho, Jennifer MacKenzie, Jagdeep Walia, Michael Geraghty, Graeme Smith, Julie Nedvidek, Andrea Guerin

**Affiliations:** ^1^ Faculty of Arts and Science Queen's University Kingston Ontario Canada; ^2^ Department of Pediatrics, Division of Medical Genetics Kingston General Hospital Kingston Ontario Canada; ^3^ Department of Pediatrics McMaster University Hamilton Ontario Canada; ^4^ Division of Metabolics, Department of Pediatrics Children's Hospital of Eastern Ontario Ottawa Ontario Canada; ^5^ Department of Obstetrics and Gynecology Kingston General Hospital Kingston Ontario Canada

**Keywords:** HHH syndrome, Ornithine transporter, urea cycle disorder, inborn error of metabolism, pregnancy, intrauterine growth restriction

## Abstract

Hyperornithinemia‐hyperammonemia‐homocitrullinuria (HHH) syndrome is a rare metabolic autosomal recessive urea cycle disorder. Only about 100 patients have been reported in the literature. As the population survives into reproductive years, pregnancy management becomes a new challenge for this clinicians. To our knowledge, there are less than three patients with successful pregnancies and deliveries found in the literature with no specific consensus on management or recommendations for HHH syndrome. We reviewed the current literature regarding pregnancy outcomes, combine it with our experience managing a patient through two successful pregnancies and identify a new concern of fetal intrauterine growth restriction. From this, recommendations for pregnancy management are made, including a detailed protocol for clinicians to use for disease management at delivery and in the post‐partum period.

## 1 INTRODUCTION

Hyperornithinemia‐hyperammonemia‐homocitrullinuria (HHH) syndrome (MIM 238970) is a rare autosomal recessive, urea cycle disorder.^1^ First described in 1969,^2^ HHH syndrome is caused by mutations of the *SLC5A15* gene, which codes for the mitochondrial ornithine carrier ORC1.^1^ Classical presentation of the condition consists of a combination of clinical and biochemical signs of hyperammonemia, hyperornithinemia, and urine excretion of homocitrulline.^1^ Clinical presentation includes irregular episodes of vomiting, confusion, and hepatitis‐like attacks.^1^ Patients can also demonstrate chronic clinical signs, including avoidance of protein‐rich foods, psychological disorders, ataxia, seizures, and pyramidal dysfunction.^1^

The treatment for HHH syndrome is similar to other urea cycle conditions and early diagnosis may improve clinical progression. Long‐term management entails a low‐protein diet based on ammonia levels and amino acid profiles, supplementation with citrulline or arginine, essential amino acids as required, and possibly ammonia lowering agents, such as sodium benzoate or sodium phenylbuterate.^3^ In addition, additional calories to spare protein are often required.

HHH syndrome is a rare metabolic disorder with approximately 100 patients reported in the literature.^1^ To our knowledge, there are less than three patients with successful pregnancies and deliveries found in the literature.^4^, ^5^, ^6^ We summarize the current state of knowledge regarding HHH and pregnancy, and suggest a protocol for management during metabolic decompensation (Table 1).

**Table 1:** Hyperammonemia orders to be initiated for significant metabolic decompensation

Diagnosis: HHH deficiency	
Monitoring	Seizure precautions Vital signs hourly ×4, then every 2 h ×4, then every 4 h if stable Strict ins and outs, recorded hourly Page attending physician if any of the following occur: Decline in level of consciousness Vomiting All blood work results IV or nasogastric solutions not available on ward within 60 min of patient admission
Laboratory	ABG, CBC, electrolytes (Na, K, HCO_3_, Cl), BUN, creatinine, ammonia, AST, ALT, Alk phos, bilirubin, glucose STAT Repeat ammonia and electrolytes STAT when priming infusion complete and every 4 h thereafter
Treatments	Elevate head of bed to 45° Insert NG tube STAT
Intravenous	Start 2 IV lines STAT 10% dextrose solution at 100 mL/h to run in each line until medications arrive
Medications	Ondansetron 8 mg IV STAT, then 4 mg IV every 8 × 48 h, then 4 mg IV every 8 h as needed IV solution #1 STAT (run in line #1):
Doses	Loading Dose AMMONUL: 55 mL/m^2^ plus arginine HCl: 250 mg/kg in 10% dextrose Infuse loading dose over 4 h using an infusion pump When loading dose completed, start maintenance dose
	Maintenance Dose AMMONUL: 55 mL/m^2^ plus arginine HCl: 250 mg/kg in 10% dextrose Infuse maintenance dose over 24 h continuously using an infusion pump IV solution #2 STAT (run in Line #2): lipids 20% 500 mL to infuse continuously over 24 h = 21 mL/h Please note lipids 20% can be given via peripheral IV
Diet	__Usual Diet___ g_protein/24 h (from food) *OR* __ Decreased Protein Diet: ___ protein/24 h (from food) *OR* __ Enteral feeds *OR* __NPO except for meds and sips of H_2_O
	SUPPLEMENTS
	_____ Essential Amino Acid Mix 10 g three times a day (24 g/protein/24 h) *OR* _____ Essential Amino Acid Mix ___ g three times a day (___ g/protein/24 h)
	If able to take oral diet please give High Energy juices (with polycose added) and Duocal 250 mL three times per day mixed at 1 kcal/mL concentration. Order High Energy Low Protein diet at set protein restriction. Please give ad lib ginger ale, regular sweetened beverages (nondiet).
	Calculation of total calories in first 24 h:
	2 × 1 L 10% dextrose × 340 kcal/L = 680 kcal 500 mL 20% lipids × 2000 kcal/L = 1000 kcal Duocal 50 g + 250 mL water three times a day = 738 kcal 3 Tbsp Polycose powder + 250 mL juice(three times a day) = 565 kcal Total kcal = 2983 kcal
	If unable to take fluids orally but can tolerate enteral feeds please order Duocal 1 kcal/mL via nasogastric feeding initiating at 30 mL/h.
	If unable to tolerate enteral feeds run IV dextrose and 20% intralipids as above with consult metabolic dietitian to determine final TPN requirements (protein free).

## 2 CASE REPORT

The patient is a 33‐year‐old woman of French‐Canadian descent. She was first diagnosed with HHH syndrome at the age of five. Genetic testing of *SLC25A15* revealed two pathogenic mutations, c.562_564del (p.Phe188del) and c.671G > A (p.Trp224*). She struggled with compliance on a low‐protein diet supplemented with amino acids and oral agents to regulate ammonia levels. Prior to pregnancy, she had no spasticity, ataxia, or seizures. She did have longstanding concerns relating to memory, presumably from previous episodes of hyperammonemia. MRI of the brain was normal. During times of illness, the patient developed hyperammonemia, with ataxic gait, extreme fatigue, and incoherent speech.

### 2.1 First pregnancy

At the age of 29, the patient successfully conceived using clomiphene citrate after a diagnosis of polycystic ovarian syndrome. Prior to pregnancy, the patient's partner underwent genetic testing and was not found to be a carrier for HHH syndrome. The patient was prescribed pyridoxine/doxylamine at the onset of vomiting in the first trimester. Prenatal ultrasounds were normal. During the first trimester, she did experience illness with ensuing weight loss, nausea, and hyperammonemia. She was followed up closely by a metabolic dietician, with biweekly to monthly plasma amino acids and ammonia measurements. She was unable to meet protein goals of 0.8 g protein/kg (approximately 50 g/day) nor adequate caloric intake. She was prescribed calorie supplements, which she took variably. She reported low appetite but was able to take her prenatal vitamin, iron, calcium, and vitamin D supplements. By third trimester, she was better able to meet calorie and protein goals and gained 14.8 kg by the 38th week. The patient was maintained on sodium phenylbutyrate 5.5 g four times a day, citrulline 2.5 g four times a day, and carnitine 350 mg four times a day. The patient was admitted at 38 weeks of gestation and underwent elective Caesarian section to reduce the risk of hyperammonemic crisis using the attached protocol (Table 1). Postpartum, the patient was admitted to the ICU for 24 hours to monitor her blood ammonia levels, to which remained in the normal range. The baby was born with birth weight of 3030 g (15‐50th centile), length of 47 cm (10‐50th centile), and head circumference of 33.5 cm (50th centile). APGAR scores were 9 and 9. At the age of 5 years, she had typical growth and development.

### 2.2 Second pregnancy

At the age of 32, the patient conceived her second child spontaneously. Again she was monitored throughout the pregnancy by a metabolic dietician. Medications at that time included sodium phenylbuterate 5 g four times a day, citrulline 8.6 g three times a day, ferrous sulfate 300 mg daily, carnitine 330 mg three times a day, essential amino acids 1 tablespoon three times a day, and calcium, vitamin D, and prenatal vitamin supplements. Complications of her HHH syndrome arose prior to the second trimester. The patient was admitted for hyperammonemia, with ammonia levels recorded of 295 μmol/L (9‐45 μmol/L) and was treated as a metabolic emergency having shown physical symptoms of nausea, lethargy, and respiratory alkalosis. She was treated using the protocol in Table 1. Ammonia levels were monitored, and she was prescribed a diet consisting of 21 g of amino acid supplements and 30 g of protein. With this protocol, the patient's ammonia levels decreased. Upon discharge, the patient was prescribed a 24‐hour diet of 30 g of natural protein and 21 g of amino acid supplement. She again had issues with appetite and oral intake throughout the pregnancy, but was able to maintain close to 0.8 g/kg protein from a combination of food and amino acids. She also had difficulty maintaining energy intake and was on a combination of carbohydrate and fat modules for additional calories. Total weight gain was similar to the previous pregnancy of 14 kg by 35 weeks’ gestation.

At 30 weeks’ gestation, fetal ultrasound was concerning for intrauterine growth restriction, which ultimately led to a repeat Caesarian section at 35 weeks and 1 day gestation. Ammonia levels remained normal during and after delivery. The female infant's birth weight was 1580 g (3rd centile), with the length of 39 cm (<3rd centile) and head circumference of 31 cm (10‐50th centile). APGAR scores were 7 and 8. The child remained in the NICU due to prematurity and low‐birth weight. There was no evidence of HHH syndrome and newborn screening was normal. At 2 years of age, the child was diagnosed with autism and speech delay.

## 3 LITERATURE SEARCH

NCBI database was searched using the keywords *hyperornithinemia hyperammonemia and homocitrullinuria pregnancy, HHH pregnancy, HHH syndrome,* and *HHH female patients pregnancy*.

## 4 RESULTS

Three reports were identified. All findings from the three reports as well as from our patient are summarized in Table 2.

**Table 2:** Summary of reported pregnancies with HHH syndrome

Ref.	Patient age	Gestation age at delivery(wk)	Ornithine (umol/L)	Citrulline (umol/L)	Ammonia (umol/L)	Pregnancy treatment strategy	Delivery treatment strategy	Delivery outcome	Fetal outcome	Follow‐up development outcome
Gatfield et al.^4^	22	35	Unk	Unk	Unk	Diet lacking of high protein foods	Protein intake of 1.0 g/kg/h	Normal	Normal	Normal
Kim et al.^5^ (Baby 1)	18	Full term	357 (during delivery)	Unk	521 (coma) 175 (postpartum)	Protein intake of 40 g/d	Cesarean‐section	Elevated blood ammonia, ornithine, and glutamine levels postpartum	IUGR	Normal
Kim et al.^5^ (Baby 2)	Unk	Unk	302 (during delivery)	Unk	Unk	Unk	Cesarean‐section	Elevated blood ammonia and ornithine levels postpartum	Normal	Normal
Kim et al.^5^ (Baby 3)	31	Unk	Unk	Unk	Unk	Unk	Cesarean‐section	Seizure	Required mechanical ventilation	Unk
Wong et al.^6^	24	39	75.6	Unk	110‐140	Protein restriction, lactulose, arginine	Unknown mode of delivery protein intake of 1.2‐1.3 g/kg/d	Normal	Normal	Normal
Our patient (Baby 1)	29	38	94	12	10‐32	Protein monitoring, sodium phenylbuterate citrulline, arginine	Caesarian section, protein restriction, ICU stay for observation, ammonia level monitoring intra and postpartum	Normal	Normal	Normal
Our patient (Baby 2)	32	35	253	24	295‐76	Protein monitoring, sodium phenylbuterate, citrulline, arginine	Caesarian section, protein restriction, ICU stay for observation, ammonia level monitoring intra and postpartum	Normal	IUGR	Speech delay

### 4.1 Patient 1

Patient 1 was a 22‐year‐old woman.^4^ The patient demonstrated severe feeding problems during infancy and was hospitalized three times for feeding issues. The patient had no history of developmental disability or seizures. The child of the patient was born at 35 weeks’ gestation. Delivery method was unreported. During labor, a protein intake of 1.0 g/kg/h was maintained. The infant's birth weight was 2150 g (10th‐50th centile), with normal length and head circumference. The infant was prescribed a diet consisting of milk formula with 3.0 g of protein/kg/24 h, and demonstrated normal blood ornithine levels at 2 weeks of age, and normal development at 6 months of age upon follow‐up.

### 4.2 Patient 2

Patient 2 was a 31‐year‐old woman of Salvadoran descent, who delivered three children.^5^ At 4 years of age, the patient first presented episodes of seizures and abnormal neurological findings and was diagnosed with HHH syndrome. The patient had developmental disability, manifested by expressive language and attention problems determined upon neuropsychiatric evaluation, and significant myopia. The patient was prescribed a diet consisting of protein restriction to 1.5 g/kg/d during childhood. The patient's weight was at the 25th percentile with height less than the 3rd percentile.

The patient's first pregnancy occurred at 18 years old. She had episodes of nausea and dizziness. She was maintained on a low‐protein diet in the first trimester, but mild hyperammonemia was detected during the 11th and 12th week of pregnancy; as a result, protein intake was further restricted to 40 g/d. It was unclear if the patient was compliant or not. At 22 weeks gestation, the patient developed seizures, and was initiated with carbamazapine. Brain imaging showed multiple small calcifications of unreported cause, with normal cerebral angiography. The child was born at full term, via Cesarean‐section. The baby girl had intrauterine growth restriction (weight <3rd percentile). Although the baby girl remained small, follow‐up recorded a normal development at her 2 years of age.

Very little is described for the course of her second and third pregnancy. The second child was born via Caesarian section. A moderately elevated ornithine concentration (302 μM/L) was measured in the cord blood, but the levels decreased to normal in the newborn's blood 24 hours after birth. The patient's blood ammonia levels increased 24 hours postpartum and were given a treatment consisting of oral sodium benzoate and intravenous arginine. The patient responded well to the treatment. Follow‐up showed normal growth and development for the child at 10 months of age.

The third child was born via repeat Cesarean section and had transient respiratory distress which required mechanical ventilation. No further information was given regarding the outcome of this child.

Upon follow‐up, the patient was noted to have been prescribed more anticonvulsants, and had significant weight loss from 95 to 50 lb. The patient died suddenly at the age of 31 years and 9 months, with an autopsy revealing two firm nodules in the brain, one each in the left parietal and frontal lobes with no determined cause of death.

### 4.3 Patient 3

Patient 3 was a 24‐year‐old woman with HHH.^6^ Clinical picture included ataxia, tremor, seizure, developmental delay (IQ of 65), and abnormal electroencephalogram. The report notes at diagnosis that the patient's blood ammonia level was >300 μg/dL (*N* < 80), ornithine levels were 71‐86 μM/dL (*N* < 15), and homocitrulline levels were 510‐643 μM/24 h. The patient was prescribed a diet consisting of lactulose, arginine, and protein restriction, which resulted in a reduction on blood ammonia levels to 110‐140 μg/dL and clinical improvement.

The patient was found to be pregnant at 8 weeks’ gestation, with blood ammonia levels of 120 μg/dL and ornithine levels of 75.6 μM/dL.^6^ The dietary protein intake was increased to 1.2‐1.3 g/kg/d. A healthy male infant was delivered at 39 weeks’ gestation. The mode of delivery was not disclosed. During the delivery, maternal ammonia levels varied from 74 to 215 μg/dL. Upon follow‐up at 16 months of age, the child's Bayley Scales showed normal development, and at age 5, the child had an IQ of 130.

## 5 DISCUSSION

### 5.1 Protein restriction during pregnancy

Protein guidelines for pregnancy in normal woman vary between DRI of 1.1 g/kg/d (for second half of pregnancy)^7^ and 1.2‐1.52 g/kg/d for early to late gestation.^8^ In pregnant women with urea cycle disorders, who are known to be averse to dietary protein often complicated with anorexia and nausea, and have noncompliance/inability to take ammonia scavenging medications, protein intake needs to be closely monitored to ensure normal amino acid profiles, prevent catabolism/metabolic decompensation, and avoid hyperammonemia. This is balanced with ensuring adequate nonprotein calories. Frequent laboratory monitoring of ammonia and plasma amino acids is recommended to adjust the amount of protein to maintain amino acids in the normal range.^9^, ^10^

Across all management plans, patients were prescribed varying dietary protein restriction, although it is difficult to quantify exactly recommended grams of protein per kilogram per patient. It varied from 40 to 25 g of dietary protein per day (our patient^5^). Häberle et al.^3^ suggests using the FAO/WHO/UNU recommendations for guiding protein requirements during pregnancy. However, we found that she was adverse to increasing natural protein particularly in the early weeks of her first pregnancy when she was ill and were never able to achieve the level suggested. Prior to pregnancy, although prescribed 25 g natural protein and 25 g amino acid supplement, she was only eating 20 g natural protein and nonadherent to citrulline, amino acids, and medications. Amino acid levels going into pregnancy were thus low. She did try to implement many of the nutrition guidelines given but found her eating pattern difficult to change despite good intent and the motivation for a healthy baby and did take vitamin supplements. Gastrointestinal symptoms in patients with urea cycles disorders are well described^11^ and represent a significant challenge for adequate protein intake in all stages of pregnancy.

Weekly or two week plasma amino acid and ammonia measurements are ideal for ongoing monitoring; however, we see in our case and in Kim et al.^5^ that compliance can be a challenge for patients. Protein and essential amino acid intake need to be frequently adjusted to avoid individual amino acid depletion and/or an increased waste nitrogen burden. Our multidisciplinary team including a metabolic physician, dietician, nurse, and pharmacist had intensive interventions with monthly visits and daily correspondence to help with compliance to variable effect.

### 5.2 Ammonia‐lowering agents during pregnancy

One patient^5^ was given sodium benzoate in the postpartum period. Our patient was the only one prescribed phenylbutyrate. Phenylbutyrate is converted into sodium phenylacetate in the gut, and then conjugated in the liver with glutamine to form phenylacetylglutamine. The compound is excreted in urine, removing the glycine and glutamine that could contribute to HHH.^12^ Our team felt the preferred agent was sodium benzoate. There is no literature to give guidance on the potential teratogenic effects of either medication, particularly in regard to growth restriction. Nonetheless, after extensive consultation for the ideal protocol, including consultation with Metabolic colleagues in Quebec, given the higher prevalence there due to a founder mutation, the decision was to continue prescription of phenylbutyrate as the patient was pregnant at the time the switch would have happened.

### 5.3 Management during delivery

Most deliveries were performed by Caesarian section (Kim et al., our patient). Most protocols for management of urea cycle disorders were general, and there was nothing in the literature regarding management during delivery. Therefore, we developed a management strategy in consultation of a multidisciplinary team including metabolic geneticists, maternal‐fetal‐medicine specialists, anesthetists, internal medicine, metabolic dietician, and social worker (Table 1), which is supported by Wilcox et al.^10^ Caesarian section was thought to offer the best option for optimal metabolic control before, during, and after delivery. This is congruent with the recommendation by Häberle et al.,^3^ which states the patient should be well and surgery planned. We also switched the patient to IV glucose and she was the first patient of the day. Many of the postoperative recommendations from Häberle et al.^3^ and Wilcox^10^ were met; however, our protocol was more discrete in terms of clear timing of blood work and vitals and it gave more guidance on dietary management postoperatively. Clear limits for ammonia were set for the initiation of intravenous ammonia scavengers, and the pediatrics team was made aware for any potential complications in the baby, steps of which have not been described elsewhere.

### 5.4 Fetal outcomes

Although five of the seven infants (71%) were of normal weight, the remaining two (29%) had growth restriction (Kim et al., our patient). This proportion is much higher than the general population, which is estimated at 3%‐7%.^13^ It is noted that the results pertain to a very small sample size. Protein was monitored carefully during both of our patient's pregnancies to optimize fetal growth. It is not known when the growth restriction presented in two patients. However, the presentation in our patient at 30 weeks, which is not a routine point for obstetrical ultrasound, would suggest that additional ultrasound monitoring is necessary. However, given the complex picture of dietary compliance, the underlying mechanism for fetal growth restriction in relation to maternal HHH syndrome is not yet known.

## 6 CONCLUSION

HHH is a rare metabolic condition with variable clinical presentation. As patients survive into reproductive age, challenges exist into appropriate management of patient and fetus to ensure favorable outcomes. We reviewed the literature for pregnancy and delivery management, and fetal outcomes for HHH syndrome. We describe our experience with patient compliance and the difficulty with balancing protein requirements and fetal growth. Although it was not the first choice, we report no teratogenic side effects to the use of sodium phenylbutyrate during two pregnancies, although further study is needed to prove safety. We suggest a detailed protocol for consideration in the management at delivery in postpartum and identify that a high rate of growth restriction for babies born to mothers with HHH syndrome. Normal developmental outcomes have been reported for most babies, which is reassuring. Given the paucity of guidance for these patients from the literature, this highlights the importance of a multidisciplinary approach tailored to the need of those pregnant with HHH syndrome.
